# Supplementary material for: A Likelihood Approach for Real-Time Calibration of Stochastic Compartmental Epidemic Models
Source: PLoS Comput Biol. 2017 Jan 17;13(1):e1005257. doi: 10.1371/journal.pcbi.1005257 (PMC5240920; doi:10.1371/journal.pcbi.1005257)
Supplement: S1 File — (TAR.GZ) [file pcbi.1005257.s014.tar.gz › HSPH_Online-SI-Revision/EnKF07/n1-extreme/n1-extreme_EnKF2_table-4.pdf]

1	newly 3 weeks spec	116.	51.67	{3., 134.}	68.2328	0.701437	newly 3 weeks cumul	225.	373.51	{146., 881.}	87.9689	0.266001	newly 5 weeks spec	137.	17.2	{0., 40.}	87.4453	1.46368	newly 5 weeks cumul	508.	421.06	{154., 986.}	50.7795	0.288326	newly 64 weeks cumul	896.	449.22	{165., 1009.}	55.8795	0.422827
2	newly 3 weeks spec	95.	13.61	{0., 37.}	89.2105	1.56167	newly 3 weeks cumul	271.	102.24	{19., 222.}	79.0332	0.728483	newly 5 weeks spec	80.	8.	{0., 8.}	95.275	2.08639	newly 5 weeks cumul	464.	120.09	{19., 234.}	81.3341	0.893729	newly 60 weeks cumul	752.	139.72	{19., 375.}	83.9947	1.06295
3	newly 3 weeks spec	74.	31.02	{3., 72.}	78.3243	0.794019	newly 3 weeks cumul	153.	229.47	{50., 528.}	96.451	0.299142	newly 5 weeks spec	91.	10.9	{0., 26.}	89.6923	1.73605	newly 5 weeks cumul	361.	256.78	{53., 607.}	71.4072	0.454728	newly 62 weeks cumul	847.	284.8	{53., 695.}	72.5006	0.698014
4	newly 3 weeks spec	66.	10.99	{0., 22.}	86.1364	1.34312	newly 3 weeks cumul	162.	127.36	{19., 274.}	65.8272	0.426006	newly 5 weeks spec	58.	2.91	{0., 7.}	94.9828	2.07938	newly 5 weeks cumul	300.	136.15	{23., 345.}	69.4433	0.606708	newly 61 weeks cumul	732.	141.33	{23., 367.}	80.9932	0.937561
5	newly 3 weeks spec	89.	11.13	{0., 12.}	93.9213	1.71574	newly 3 weeks cumul	241.	68.22	{14., 59.}	91.9087	0.982352	newly 5 weeks spec	98.	5.75	{0., 6.}	94.949	2.29798	newly 5 weeks cumul	447.	81.33	{16., 114.}	89.5011	1.1625	newly 54 weeks cumul	764.	101.29	{17., 273.}	88.216	1.32135
6	newly 3 weeks spec	46.	10.52	{0., 15.}	95.2174	1.33513	newly 3 weeks cumul	105.	77.	{7., 88.}	95.219	0.595715	newly 5 weeks spec	44.	4.02	{0., 5.}	94.3182	1.9772	newly 5 weeks cumul	246.	87.07	{8., 104.}	90.4919	0.877388	newly 59 weeks cumul	557.	93.81	{8., 116.}	89.2262	1.1725
7	newly 3 weeks spec	75.	40.12	{1., 106.}	75.6	0.790604	newly 3 weeks cumul	185.	349.63	{84., 942.}	118.081	0.300572	newly 5 weeks spec	81.	12.09	{0., 36.}	86.8519	1.51164	newly 5 weeks cumul	355.	382.61	{101., 979.}	73.2648	0.32806	newly 63 weeks cumul	753.	409.36	{116., 979.}	63.8858	0.482859
8	newly 3 weeks spec	76.	17.27	{0., 31.}	88.3026	1.14936	newly 3 weeks cumul	224.	101.95	{15., 135.}	93.6652	0.813823	newly 5 weeks spec	72.	7.58	{0., 10.}	93.8333	1.75974	newly 5 weeks cumul	382.	121.22	{18., 171.}	90.1885	0.944985	newly 57 weeks cumul	642.	139.07	{18., 220.}	88.1854	1.08207
9	newly 3 weeks spec	86.	51.62	{3., 122.}	65.2558	0.618481	newly 3 weeks cumul	199.	365.82	{151., 890.}	99.1055	0.287793	newly 5 weeks spec	96.	17.35	{0., 35.}	83.0729	1.28204	newly 5 weeks cumul	400.	413.42	{166., 939.}	51.99	0.260329	newly 64 weeks cumul	731.	439.56	{173., 957.}	53.5103	0.389738
10	newly 3 weeks spec	73.	14.14	{0., 18.}	94.1644	1.44799	newly 3 weeks cumul	217.	68.49	{5., 118.}	88.0046	0.923331	newly 5 weeks spec	62.	9.15	{0., 14.}	95.4355	1.86	newly 5 weeks cumul	362.	89.46	{6., 140.}	89.4641	1.05625	newly 57 weeks cumul	751.	116.52	{6., 197.}	88.7617	1.27008
11	newly 3 weeks spec	88.	41.26	{0., 94.}	74.8864	0.874392	newly 3 weeks cumul	241.	361.42	{91., 922.}	89.3859	0.26982	newly 5 weeks spec	81.	11.2	{0., 32.}	87.1852	1.55163	newly 5 weeks cumul	416.	394.08	{107., 956.}	69.	0.336785	newly 63 weeks cumul	680.	415.27	{124., 956.}	62.9632	0.437634
12	newly 3 weeks spec	65.	53.2	{4., 129.}	67.6615	0.517196	newly 3 weeks cumul	159.	366.09	{123., 869.}	141.906	0.336918	newly 5 weeks spec	109.	17.21	{0., 39.}	84.8532	1.37588	newly 5 weeks cumul	387.	413.76	{143., 980.}	56.2119	0.265915	newly 64 weeks cumul	698.	440.55	{150., 1002.}	52.8811	0.37356
13	newly 3 weeks spec	49.	17.23	{1., 23.}	90.5102	0.953603	newly 3 weeks cumul	125.	96.88	{15., 94.}	103.056	0.570179	newly 5 weeks spec	41.	7.59	{0., 15.}	89.2439	1.4483	newly 5 weeks cumul	222.	115.12	{16., 131.}	94.2432	0.710187	newly 57 weeks cumul	659.	137.36	{16., 206.}	86.519	1.04141
14	newly 3 weeks spec	113.	11.85	{0., 25.}	91.885	1.53588	newly 3 weeks cumul	293.	185.59	{25., 403.}	74.8089	0.588787	newly 5 weeks spec	92.	2.89	{0., 5.}	97.163	2.25777	newly 5 weeks cumul	501.	194.4	{26., 414.}	74.1916	0.738841	newly 61 weeks cumul	659.	197.58	{26., 418.}	77.0319	0.835872
15	newly 3 weeks spec	61.	13.63	{0., 43.}	88.7705	1.51756	newly 3 weeks cumul	196.	70.55	{6., 140.}	86.75	0.905265	newly 5 weeks spec	65.	5.83	{0., 14.}	92.3538	2.12397	newly 5 weeks cumul	338.	84.18	{6., 262.}	85.3846	1.06762	newly 56 weeks cumul	615.	102.7	{6., 333.}	86.3707	1.26596
16	newly 3 weeks spec	79.	39.91	{0., 77.}	73.9114	0.804737	newly 3 weeks cumul	198.	331.79	{84., 858.}	99.1364	0.294496	newly 5 weeks spec	51.	10.9	{0., 23.}	84.3137	1.33349	newly 5 weeks cumul	335.	362.69	{95., 973.}	68.003	0.314655	newly 63 weeks cumul	677.	380.51	{100., 973.}	64.6809	0.48548
17	newly 3 weeks spec	73.	26.03	{0., 64.}	96.1507	1.41471	newly 3 weeks cumul	179.	187.25	{19., 734.}	114.821	0.594331	newly 5 weeks spec	61.	8.21	{0., 39.}	89.3934	1.89258	newly 5 weeks cumul	321.	210.79	{20., 854.}	94.3956	0.720428	newly 60 weeks cumul	748.	229.92	{21., 877.}	78.2941	0.927884
18	newly 3 weeks spec	100.	34.83	{0., 71.}	75.51	0.921762	newly 3 weeks cumul	211.	288.29	{67., 672.}	82.8009	0.303808	newly 5 weeks spec	103.	11.4	{0., 24.}	89.8058	1.62838	newly 5 weeks cumul	426.	319.71	{74., 731.}	65.4155	0.411932	newly 63 weeks cumul	855.	344.87	{88., 806.}	68.9298	0.613635
19	newly 3 weeks spec	67.	59.07	{4., 141.}	60.8507	0.493653	newly 3 weeks cumul	165.	381.58	{147., 875.}	139.212	0.330818	newly 5 weeks spec	77.	21.44	{0., 50.}	78.	1.15055	newly 5 weeks cumul	334.	438.52	{172., 953.}	57.8743	0.212673	newly 64 weeks cumul	820.	470.91	{177., 1024.}	52.65	0.37546
20	newly 3 weeks spec	98.	8.62	{0., 13.}	94.5306	1.80899	newly 3 weeks cumul	274.	58.69	{5., 75.}	88.4051	0.998082	newly 5 weeks spec	84.	3.28	{0., 7.}	96.0952	2.159	newly 5 weeks cumul	458.	67.13	{5., 91.}	90.8231	1.14676	newly 57 weeks cumul	701.	74.76	{5., 133.}	91.7347	1.28775
21	newly 3 weeks spec	44.	19.42	{0., 47.}	97.	1.2944	newly 3 weeks cumul	115.	146.16	{12., 327.}	121.183	0.508541	newly 5 weeks spec	43.	7.6	{0., 15.}	93.8605	1.90153	newly 5 weeks cumul	219.	164.62	{12., 562.}	97.9635	0.668196	newly 60 weeks cumul	654.	181.45	{12., 840.}	83.1789	1.0098
22	newly 3 weeks spec	79.	11.25	{0., 25.}	88.8987	1.41952	newly 3 weeks cumul	241.	168.6	{23., 408.}	72.249	0.499526	newly 5 weeks spec	63.	2.29	{0., 4.}	96.3651	2.28651	newly 5 weeks cumul	388.	175.48	{24., 423.}	68.8351	0.61713	newly 61 weeks cumul	683.	178.08	{24., 423.}	76.7526	0.824658
23	newly 3 weeks spec	72.	39.78	{0., 80.}	71.9167	0.764704	newly 3 weeks cumul	168.	282.16	{82., 533.}	89.5952	0.239321	newly 5 weeks spec	79.	13.43	{0., 34.}	86.443	1.43793	newly 5 weeks cumul	346.	317.92	{100., 534.}	58.0867	0.283513	newly 63 weeks cumul	679.	346.43	{111., 804.}	63.9779	0.468579
24	newly 3 weeks spec	106.	54.19	{2., 140.}	67.6887	0.76626	newly 3 weeks cumul	236.	377.32	{116., 902.}	89.0593	0.322042	newly 5 weeks spec	137.	17.42	{0., 46.}	87.2847	1.53603	newly 5 weeks cumul	531.	424.76	{136., 1040.}	54.4369	0.373401	newly 64 weeks cumul	842.	449.72	{136., 1057.}	56.5582	0.481235
25	newly 3 weeks spec	63.	10.91	{0., 26.}	85.381	1.37302	newly 3 weeks cumul	139.	128.64	{20., 291.}	71.3381	0.400482	newly 5 weeks spec	68.	3.28	{0., 7.}	95.1765	2.12481	newly 5 weeks cumul	276.	137.4	{24., 299.}	70.6232	0.592075	newly 61 weeks cumul	696.	142.78	{24., 358.}	80.4224	0.926879
26	newly 3 weeks spec	131.	36.45	{0., 74.}	75.916	1.01218	newly 3 weeks cumul	280.	312.09	{104., 696.}	59.9393	0.256005	newly 5 weeks spec	153.	10.01	{0., 28.}	93.4575	1.84503	newly 5 weeks cumul	589.	340.2	{114., 831.}	60.9779	0.426414	newly 63 weeks cumul	887.	354.96	{131., 843.}	65.177	0.544849
27	newly 3 weeks spec	61.	10.99	{0., 16.}	96.0164	1.86432	newly 3 weeks cumul	160.	92.19	{5., 86.}	101.094	0.797815	newly 5 weeks spec	54.	3.73	{0., 5.}	96.0556	2.22313	newly 5 weeks cumul	277.	102.18	{5., 118.}	96.2166	0.979672	newly 58 weeks cumul	659.	107.9	{5., 138.}	90.4036	1.28911
28	newly 3 weeks spec	77.	30.11	{1., 75.}	81.7792	0.947652	newly 3 weeks cumul	250.	231.93	{35., 503.}	82.364	0.440449	newly 5 weeks spec	99.	10.27	{0., 25.}	89.9091	1.8475	newly 5 weeks cumul	460.	258.38	{37., 628.}	73.5217	0.579139	newly 62 weeks cumul	803.	281.45	{37., 867.}	73.812	0.744549
29	newly 3 weeks spec	145.	13.4	{0., 19.}	92.8414	1.71408	newly 3 weeks cumul	304.	88.46	{9., 121.}	89.7763	0.968599	newly 5 weeks spec	113.	3.72	{0., 7.}	96.708	2.33777	newly 5 weeks cumul	568.	99.4	{10., 126.}	89.2817	1.17221	newly 59 weeks cumul	875.	108.68	{10., 126.}	88.6217	1.31935
30	newly 3 weeks spec	52.	8.84	{0., 21.}	89.6154	1.50021	newly 3 weeks cumul	145.	93.69	{6., 152.}	102.338	0.767844	newly 5 weeks spec	41.	3.	{0., 6.}	95.2195	2.19144	newly 5 weeks cumul	245.	101.74	{7., 243.}	93.2816	0.929569	newly 54 weeks cumul	398.	107.47	{7., 255.}	88.5151	1.09136
31	newly 3 weeks spec	115.	37.88	{0., 73.}	76.8174	0.976219	newly 3 weeks cumul	258.	282.68	{90., 507.}	61.3953	0.276654	newly 5 weeks spec	94.	11.2	{0., 32.}	88.4043	1.63493	newly 5 weeks cumul	471.	313.4	{106., 508.}	59.6518	0.398135	newly 63 weeks cumul	796.	335.62	{119., 833.}	65.4749	0.554098
32	newly 3 weeks spec	106.	36.53	{0., 71.}	76.3302	1.02005	newly 3 weeks cumul	229.	292.14	{79., 706.}	75.4585	0.288764	newly 5 weeks spec	128.	11.98	{0., 34.}	90.6563	1.73306	newly 5 weeks cumul	487.	323.39	{102., 776.}	65.7433	0.437619	newly 63 weeks cumul	773.	350.99	{104., 798.}	66.1229	0.551121
33	newly 3 weeks spec	60.	17.8	{0., 33.}	91.5333	1.24391	newly 3 weeks cumul	152.	103.83	{6., 233.}	101.428	0.743144	newly 5 weeks spec	78.	4.71	{0., 16.}	94.0897	2.03622	newly 5 weeks cumul	313.	116.9	{7., 353.}	89.9361	0.946828	newly 59 weeks cumul	799.	127.2	{7., 439.}	86.1527	1.25603
34	newly 3 weeks spec	85.	24.56	{2., 50.}	80.9176	0.930069	newly 3 weeks cumul	234.	196.39	{37., 429.}	73.765	0.423656	newly 5 weeks spec	96.	8.38	{0., 18.}	92.2083	1.84299	newly 5 weeks cumul	427.	217.9	{37., 486.}	71.6206	0.574565	newly 62 weeks cumul	764.	241.15	{42., 490.}	76.5092	0.766001
35	newly 3 weeks spec	73.	34.29	{1., 80.}	71.9589	0.775418	newly 3 weeks cumul	181.	302.51	{76., 715.}	97.5414	0.283248	newly 5 weeks spec	79.	10.73	{0., 25.}	89.3291	1.5247	newly 5 weeks cumul	361.	331.87	{96., 778.}	64.8283	0.338709	newly 63 weeks cumul	749.	352.91	{96., 848.}	66.4633	0.538937
36	newly 3 weeks spec	81.	27.12	{0., 112.}	93.4568	1.42164	newly 3 weeks cumul	195.	184.7	{15., 640.}	102.	0.568061	newly 5 weeks spec	83.	10.69	{0., 34.}	91.5783	2.0263	newly 5 weeks cumul	376.	212.14	{20., 835.}	86.5213	0.722009	newly 60 weeks cumul	755.	233.24	{26., 885.}	77.7907	0.911663
37	newly 3 weeks spec	78.	10.19	{0., 41.}	87.2692	1.62569	newly 3 weeks cumul	218.	88.65	{10., 355.}	89.344	0.863685	newly 5 weeks spec	65.	2.22	{0., 7.}	96.5846	2.21171	newly 5 weeks cumul	380.	95.02	{10., 376.}	84.2526	1.01966	newly 55 weeks cumul	728.	97.36	{10., 376.}	86.6264	1.25452
38	newly 3 weeks spec	93.	16.89	{0., 35.}	90.3118	1.5311	newly 3 weeks cumul	234.	136.96	{17., 296.}	84.359	0.63024	newly 5 weeks spec	114.	9.54	{0., 17.}	95.0877	2.24835	newly 5 weeks cumul	462.	157.55	{18., 497.}	80.4134	0.817856	newly 60 weeks cumul	754.	176.78	{19., 664.}	80.1459	0.969055
39	newly 3 weeks spec	89.	36.34	{0., 74.}	72.7191	0.890763	newly 3 weeks cumul	179.	312.2	{101., 663.}	95.5642	0.265974	newly 5 weeks spec	117.	9.71	{0., 26.}	91.7009	1.77256	newly 5 weeks cumul	414.	340.68	{103., 824.}	56.1159	0.31169	newly 63 weeks cumul	855.	355.15	{113., 834.}	64.3754	0.525819
40	newly 3 weeks spec	70.	14.16	{0., 23.}	88.7714	1.28924	newly 3 weeks cumul	153.	201.06	{23., 428.}	101.281	0.42432	newly 5 weeks spec	66.	3.23	{0., 7.}	95.6515	2.19601	newly 5 weeks cumul	299.	210.58	{25., 428.}	77.3512	0.559032	newly 61 weeks cumul	575.	215.1	{25., 450.}	74.7096	0.751234
41	newly 3 weeks spec	45.	12.15	{0., 29.}	92.3333	1.43772	newly 3 weeks cumul	135.	78.72	{8., 100.}	98.4593	0.756153	newly 5 weeks spec	55.	5.74	{0., 9.}	93.5636	2.03391	newly 5 weeks cumul	248.	92.6	{8., 157.}	93.2984	0.941745	newly 56 weeks cumul	562.	109.26	{8., 229.}	88.8541	1.21969
42	newly 3 weeks spec	88.	58.	{4., 122.}	60.6136	0.533367	newly 3 weeks cumul	235.	377.78	{141., 859.}	82.6809	0.261258	newly 5 weeks spec	105.	18.49	{0., 50.}	82.3905	1.24618	newly 5 weeks cumul	462.	427.76	{158., 990.}	49.9697	0.266659	newly 64 weeks cumul	862.	453.74	{169., 1069.}	55.2645	0.416597
43	newly 3 weeks spec	82.	36.98	{0., 69.}	72.5366	0.81445	newly 3 weeks cumul	219.	304.38	{101., 678.}	71.4977	0.237752	newly 5 weeks spec	75.	11.39	{0., 35.}	85.72	1.44182	newly 5 weeks cumul	390.	335.45	{107., 816.}	55.2846	0.29306	newly 63 weeks cumul	860.	356.07	{125., 847.}	64.8058	0.527594
44	newly 3 weeks spec	86.	6.91	{0., 17.}	91.9651	1.58221	newly 3 weeks cumul	240.	141.68	{13., 391.}	75.2833	0.615934	newly 5 weeks spec	76.	1.32	{0., 4.}	98.2632	2.33344	newly 5 weeks cumul	408.	145.72	{13., 404.}	72.1324	0.754697	newly 61 weeks cumul	762.	146.96	{13., 404.}	81.6562	0.998614
45	newly 3 weeks spec	79.	55.08	{5., 133.}	62.1772	0.568164	newly 3 weeks cumul	203.	364.78	{147., 866.}	96.2069	0.267873	newly 5 weeks spec	105.	17.09	{0., 39.}	83.7238	1.27938	newly 5 weeks cumul	422.	412.02	{158., 891.}	50.6303	0.249197	newly 64 weeks cumul	886.	436.9	{169., 952.}	56.763	0.430621
46	newly 3 weeks spec	93.	8.4	{0., 18.}	90.9677	1.53256	newly 3 weeks cumul	223.	136.53	{19., 382.}	72.6054	0.540169	newly 5 weeks spec	79.	1.55	{0., 5.}	98.038	2.29736	newly 5 weeks cumul	409.	141.41	{19., 388.}	72.7115	0.71612	newly 61 weeks cumul	815.	143.65	{19., 390.}	83.1988	0.990371
47	newly 3 weeks spec	63.	29.17	{2., 70.}	80.4921	0.811604	newly 3 weeks cumul	164.	230.03	{37., 602.}	95.7256	0.318999	newly 5 weeks spec	67.	10.04	{0., 24.}	90.6269	1.69503	newly 5 weeks cumul	308.	255.88	{54., 648.}	76.8377	0.432678	newly 62 weeks cumul	785.	284.39	{55., 751.}	73.3541	0.689874
48	newly 3 weeks spec	60.	26.85	{2., 66.}	81.7167	0.885719	newly 3 weeks cumul	141.	219.76	{41., 453.}	105.915	0.31647	newly 5 weeks spec	68.	8.85	{0., 15.}	91.25	1.82198	newly 5 weeks cumul	280.	242.57	{43., 578.}	81.975	0.45185	newly 62 weeks cumul	672.	265.32	{43., 725.}	74.875	0.69401
49	newly 3 weeks spec	96.	57.63	{0., 150.}	67.5313	0.739137	newly 3 weeks cumul	261.	392.17	{128., 895.}	78.1877	0.276675	newly 5 weeks spec	115.	18.93	{0., 46.}	84.0609	1.45352	newly 5 weeks cumul	493.	444.15	{135., 1011.}	54.3387	0.317062	newly 64 weeks cumul	853.	469.95	{158., 1058.}	54.6319	0.433886
50	newly 3 weeks spec	57.	29.98	{0., 86.}	83.0175	0.916808	newly 3 weeks cumul	158.	243.25	{23., 548.}	111.171	0.427101	newly 5 weeks spec	54.	9.71	{0., 29.}	87.8333	1.60338	newly 5 weeks cumul	272.	269.48	{24., 771.}	86.9191	0.510345	newly 62 weeks cumul	579.	292.82	{26., 885.}	74.7668	0.697087
